# Dexmedetomidine-based intravenous anesthesia of a pediatric patient with glucose-6-phosphate dehydrogenase (G6PD) deficiency: A case report

**DOI:** 10.1097/MD.0000000000006986

**Published:** 2017-05-26

**Authors:** Nanae Takahashi, Takashi Ogawa, Zen’ichiro Wajima, Akibumi Omi

**Affiliations:** a Department of Anesthesiology; b Department of Oral and Maxillofacial Surgery, Tokyo Medical University Hachioji Medical Center, Tokyo, Japan.

**Keywords:** anesthesia, case report, dexmedetomidine, glucose-6-phosphate dehydrogenase (G6PD) deficiency, pediatric

## Abstract

**Rationale:**

Glucose-6-phosphate dehydrogenase (G6PD) deficiency is the most common human enzyme defect, resulting in deficits in nicotinamide adenine dinucleotide phosphate production, an important intracellular antioxidant enzyme. G6PD-deficient subjects present with a susceptibility of erythrocytes to oxidative stress and hemolysis, and should avoid drugs or stressors that have oxidative actions. Dexmedetomidine is an anesthetic agent with antioxidant actions.

**Patient concerns and diagnoses:**

A 5-year-old boy with G6PD deficiency. The patient was diagnosed with G6PD deficiency at birth. His red blood cell levels were indicating Class II G6PD activity by the World Health Organization (WHO) classification, but had no history of hemolytic anemia.

**Intraventions:**

Because of the patient's anxiety and hyperactivity prior to an operation for upper labial frenum resection, we performed perioperative management using intravenous sedation with dexmedetomidine, which provides upper airway patency and has an antioxidant action.

**Outcomes:**

There was no abnormal breathing observed during anesthesia, and arousal was smooth with stable hemodynamics. The patient had no symptoms of hemolytic anemia up to 1 week postsurgery.

**Conclusion:**

Antioxidant sedatives such as dexmedetomidine may be useful for reducing the risk of hemolysis after surgery in infant G6PD deficiency cases.

## 1 Introduction

Glucose-6-phosphate dehydrogenase (G6PD) deficiency is the most common human enzyme defect present in more than 400 million people world-wide. Patients with G6PD are resistant against malaria and predominantly live in tropical and subtropical regions such as Southeast Asia and Mediterranean countries where malaria is present.^[1]^ G6PD deficiency is caused by an X-linked recessive inheritance, and the majority of subjects who develop G6PD are males with a hemizygotic abnormality. According to the World Health Organization (WHO), 7.5% of G6PD subjects have mutant alleles, whereas 3.4% express the deficient phenotype (including all male hemizygotes, all female homozygotes, and 10% of female heterozygotes).^[2]^ By contrast, only 14% of heterozygous females were reported to exhibit deficient G6PD activity, whereas 33.3% had intermediate activity, and more than 50% had normal activity.^[3]^

G6PD is a rate-limiting enzyme of the pentose phosphate cycle, which results in production of nicotinamide adenine dinucleotide phosphate (NADPH), an important intracellular antioxidant enzyme. In patients with G6PD deficiency, the production of NADPH is impaired, resulting in susceptibility of erythrocytes to oxidative stress and ultimately hemolysis. Thus, patients with G6PD deficiency should avoid drugs with oxidative actions, fava beans, and limit stressors such as infection and fever to prevent hemolysis.^[1]^ Dexmedetomidine is an anesthetic agent with antioxidant actions.^[4,5]^ In the present study, we report a novel case of intravenous perioperative anesthesia management using dexmedetomidine for an upper labial frenum resection, in an infant patient with G6PD deficiency.

## 2 Case report

The patient was a 5-year-old boy (height, 115 cm; weight, 22 kg). He visited our Medical Centre for upper labial frenum resection, following referral from a local doctor. He was born through a normal delivery between a Japanese father and a Taiwanese mother. The grandfather (maternal side) of the patient had glucose-6-phosphate dehydrogenase (G6PD) deficiency. Both his mother and father had normal G6PD activity. G6PD enzyme activity was measured immediately after birth, and he was diagnosed with G6PD deficiency based on a marked reduction in G6PD enzyme activity. His red blood cell levels were 0.7 IU.gHb-1 (normal range, 7.61–9.81 IU.gHb-1), indicating Class II G6PD activity by the WHO classification. G6PD deficiency was diagnosed by medical records. He had no previous evidence of hemolytic anemia or similar disorder, and no comorbidity. He had a penicillin allergy and amblyopia, but no previous history such as hemolytic anemia. He had occasionally taken an over-the-counter cold medicine, but had no regular medicine use. He had not received anesthesia or sedation. He had been living normally at home, although he was prohibited from eating fava beans by his doctor. His Taiwanese grandfather (maternal side) was also G6PD deficient, but never developed hemolytic anemia. Because of his anxiety and hyperactivity before the operation, we performed perioperative management using intravenous sedation with dexmedetomidine, which provides upper airway patency^[6]^ and has an antioxidant action.^[4,5]^

On the day of anesthesia, premedication was not performed in our patient. Local xylocaine tape was placed on his forearm to relieve stress during placement of the intravenous line. The patient was slightly excited when he entered the outpatient room. His blood pressure, heart rate, and respiratory rate were 106/58 mm Hg, 108 beats per min, and 22 to 24 breaths per min, respectively. We monitored electrocardiogram, heart rate, peripheral arterial oxygen saturation, and respiratory rate during intravenous sedation. After intravenous induction of anesthesia with 2 mg of midazolam and 25 μg of fentanyl, oxygen was started at 3 L/min with a nasal cannula. Dexmedetomidine was initiated with continuous dosing, rather than a loading dose. Thus, no changes in intraoperative blood pressure were observed. Oral surface anesthesia was performed, followed by local infiltration anesthesia with 1 mL of 2% lidocaine (containing 1/80,000 of epinephrine). The operation and anesthesia was finished in 15 minutes and 35 minutes, respectively. There was no abnormal breathing observed during anesthesia, and arousal was smooth with stable hemodynamics. Cephalosporin antibiotics were administered after surgery. The child did not exhibit postoperative nausea and vomiting or agitation. The patient had no symptoms of hemolytic anemia, such fatigue, headache, or dark urine, up to 1 week post-surgery.

## 3 Discussion

Glucose-6-phosphate dehydrogenase (G6PD) deficiency is characterized by acute hemolytic crisis, for which drugs, infection, surgery, and fava beans are known triggers. Considerations for perioperative management of patients with G6PD deficiency include reducing operative stress and pain that may cause oxidative stress, selecting drugs with antioxidant actions, avoiding oxidative drugs, and limiting the potential for infection and acute hemolytic crisis.^[1]^ Forcible ventilation during general anesthesia is also thought to cause oxidative stress.^[7]^ Thus, intravenous sedation is often used to preserve spontaneous breathing, as in the present case. To prevent further deterioration of nicotinamide adenine dinucleotide phosphate (NADPH) production during perioperative management of patients with G6PD deficiency, anesthetic management should be chosen to minimize invasiveness and potential for oxidative stress. Indeed, the WHO provides a classification of drugs according to their effects on G6PD activity (Table 1). The variants of G6PD deficiency are divided into 5 classes based on the functional severity of the deficiency. Class I includes severely deficient variants associated with a chronic non-spherocytic hemolytic anemia. Class II variants have less than 10% of residual enzyme activity, but do not exhibit chronic nonspherocytic hemolytic anemia, and include the common severe Oriental variants. Class III variants are moderately deficient (10–60% residual enzyme activity), and include the common African form. Class IV variants have normal enzyme activity, whereas class V variants show increased enzyme activity.^[2]^

**Table 1:**
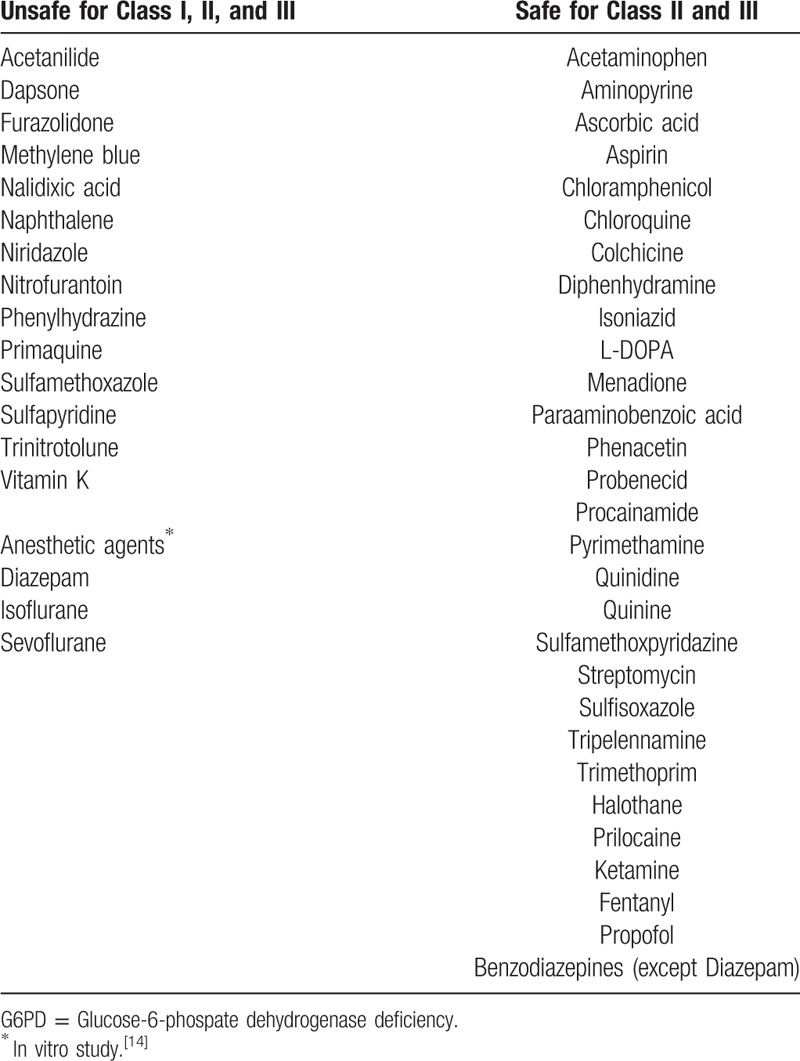
Drugs, chemicals, and anesthetic agents used in G6PD deficiency.^[13,14]^

Dexmedetomidine provides a largely natural induction of sleep, and thus is unlikely to cause respiratory suppression. In infants, anesthesia can result in airway obstruction because of their anatomically large head and tongue, as well as the frontal position of the pharynx and the long and U-shaped epiglottis. Dexmedetomidine with airway patency was reported to be a useful sedative for infants.^[8,9]^ Dexmedetomidine can also induce concurrent antioxidant, anti-inflammatory, and sedative effects. Potential side effects of dexmedetomidine include changes in blood pressure during loading and bradycardia during continuous dosing, likely because of sympathetic blockade and parasympathetic hyperactivity, as well as sinoatrial node and atrioventricular node suppression.^[10]^ Thus, particular attention to variations in the heart rate is required when using dexmedetomidine in infants.

In the present case, the stable circulatory dynamics were likely achieved by the combination of fentanyl and midazolam,^[11]^ thus avoiding the requirement for dexmedetomidine loading. Fentanyl and midazolam are reportedly safe for use in G6PD deficiency. Despite our case becoming excited when he sat on the dental unit, we obtained smooth anesthesia by pre-administration, which likely contributed to the maintenance of stable breathing and circulatory dynamics with dexmedetomidine. Dexmedetomidine loading also requires a long induction time, and there is a risk for hypotension. Thus, no changes in intraoperative blood pressure were observed in our case. In addition, as dexmedetomidine can inhibit salivary secretion, it is suitable for anesthesia management in the field of dentistry and oral surgery.^[12]^ Furthermore, dexmedetomidine can be used in cases requiring prevention of aspiration, as it does not inhibit the laryngeal reflex.^[13]^

In G6PD deficiency patients, general drug-induced hemolysis occurs at 24 to 72 hours after drug administration, while symptoms of anemia appear by 7 days after hemolysis. In addition to the symptoms of fatigue, headache, and dark red urine, hemolytic anemia during perioperative period, anemia, reduced haptoglobin levels, and elevation of bilirubin, urobilinogen, and lactate dehydrogenase levels, have been reported.^[1]^ Particular attention to evidence of hemolysis for at least 1 week after surgery is important. In the present case, there were no changes in activity of the infant, or physical symptoms indicative of hemolytic anemia, during the 1-week follow up. In our patient, we initially focused on the physical symptoms and sought to prevent discomfort, and thus performed a urine test rather than a stressful blood test. There were no physical symptoms suggestive of hemolysis. Nevertheless, for future studies, we will purchase a system capable of rapid blood sampling for a definitive diagnosis of hemolysis.

## 4 Conclusion

The use of drugs with antioxidant effects for outpatient medication, and for less invasive operative and anesthetic methods, is important to improve the safety of Glucose-6-phosphate dehydrogenase (G6PD) infant patients during the perioperative period. Intravenous sedation using dexmedetomidine was useful for surgery in our infant G6PD case.
